# How Much Information is Needed to Infer Reticulate Evolutionary Histories?

**DOI:** 10.1093/sysbio/syu076

**Published:** 2014-09-18

**Authors:** Katharina T. Huber, Leo Van Iersel, Vincent Moulton, Taoyang Wu

**Affiliations:** ^1^School of Computing Sciences, University of East Anglia, Norwich, UK, and ^2^Centrum Wiskunde & Informatica (CWI), Amsterdam, Netherlands

**Keywords:** Evolutionary tree, network reconstruction, phylogenetic network, reticulate evolution

## Abstract

Phylogenetic networks are a generalization of evolutionary trees and are an important tool for analyzing reticulate evolutionary histories. Recently, there has been great interest in developing new methods to construct rooted phylogenetic networks, that is, networks whose internal vertices correspond to hypothetical ancestors, whose leaves correspond to sampled taxa, and in which vertices with more than one parent correspond to taxa formed by reticulate evolutionary events such as recombination or hybridization. Several methods for constructing evolutionary trees use the strategy of building up a tree from simpler building blocks (such as triplets or clusters), and so it is natural to look for ways to construct networks from smaller networks. In this article, we shall demonstrate a fundamental issue with this approach. Namely, we show that even if we are given *all* of the subnetworks induced on all proper subsets of the leaves of some rooted phylogenetic network, we still do not have all of the information required to completely determine that network. This implies that even if *all* of the building blocks for some reticulate evolutionary history were to be taken as the input for any given network building method, the method might still output an incorrect history. We also discuss some potential consequences of this result for constructing phylogenetic networks.

Modern systematics assumes a tree as an integral component of the evolutionary model (Penny et al. 1992). However, genome science is also delivering a level of complexity previously under appreciated for many biological systems and organisms (e.g., Mallet 2007; Abbott et al. 2013; Liu et al. 2013; Muhlfeld et al. 2014). This growing appreciation has in turn motivated the development of phylogenetic networks (see e.g., Huson et al. 2010; Morrison 2011. These networks are a generalization of evolutionary trees and, in the broadest sense, can be any type of graph-theoretical network that is used to represent potentially complex patterns of evolutionary relationship.

Some networks, also referred to as data-display or split networks (Dress and Huson 2004; Morrison 2010), attempt only to represent bipartitions or splits in data, and the evidence these splits provide for contradictory relationships. In these networks the internal nodes usually have no explicit meaning. Such networks generalize unrooted evolutionary trees and have been used to visualize homoplasy and detect errors in human sequence data (e.g., Bandelt et al. 2000; Bandelt et al. 2001), for visualizing the support for particular bifurcating trees and hypotheses (e.g., Holland et al. 2005) and for exploring the genetic complexity of plant and animal data sets (e.g., Morrison 2005). There are numerous ways of computing these graphs (e.g., Huson and Bryant 2006). Methods essentially differ in the extent to which they visualize incompatibilities either because of the way they compute the splits and/or because of the dimensionality of the displayed network.

Other networks, also referred to as genealogical networks, are constructed to model evolutionary history wherein the evolution is suspected of being reticulate in nature. These networks, which are the focus of the present study, are typically rooted and contain internal vertices that represent hypothetical ancestors and leaves that represent taxa sampled from the data (extant or extinct). They are directed graphs with a single root vertex and leaves labeled with taxon names (see e.g., Fig. 1 and Mathematical Definitions section). They also contain no directed cycles, thus ensuring that no taxon can be a descendent of itself. In these networks, vertices with more than one parent correspond to taxa that are formed by reticulate evolutionary events such as recombination or hybridization. In particular, a rooted evolutionary tree is a special type of rooted phylogenetic network which does not represent any reticulate evolutionary events. Genealogical networks are reviewed in for example (Huson et al. 2010), and have been used to study the evolution of organisms such as plants (Marcussen et al. 2011), viruses (Visser et al. 2012), and bacteria (Kunin et al. 2005).

**Figure 1.**
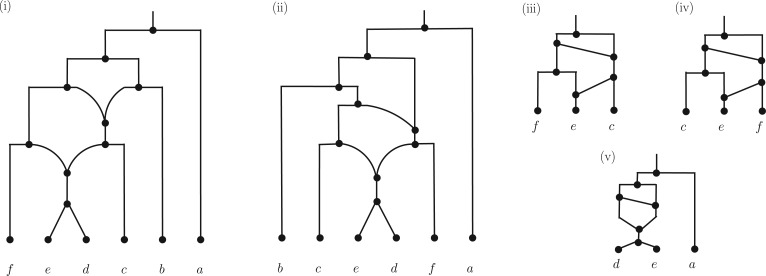
i) and ii): Two phylogenetic networks on the set of taxa {a,b,c,d,e,f}. iii) and iv): The trinets induced on the leaves c,e,f in networks in (i) and (ii), respectively. (v): The trinet induced on the leaves a,d,e by both of the networks in (i) and (ii). Here the network in (i) is the subnetwork of the network in (van Iersel et al. 2009, Fig. 10) computed from a data set of the yeast *Cryptococcus gattii*, where taxa a,b,⋯,f correspond to taxa 1,16,8,18,7 and 20, respectively, in the yeast network.

Various methods have been proposed to construct genealogical phylogenetic networks, although it is generally agreed that there is still much more to be done in this direction (see e.g., Nakhleh 2011; Bapteste et al. 2013). Many of these methods follow a strategy that is also commonly used to build evolutionary trees (e.g., to construct supertrees), namely to infer networks from building blocks such as triplets (evolutionary trees with three leaves) (Huber et al. 2011), evolutionary trees (Kelk et al. 2012; D.Huson and Scornavacca 2012), or clusters/clades (van Iersel et al. 2010). However, a fundamental issue with this strategy is that the commonly used building blocks do not necessarily determine or *encode* networks, in contrast to evolutionary trees. In other words, there can be pairs of rooted phylogenetic networks that do not represent the same evolutionary histories, but still display exactly the same building blocks (see e.g., Gambette and Huber (2012) for triplets and clusters, and Willson (2011) for evolutionary trees). For example, considering the two networks in Figure 1 (i) and (ii), the first one of which is adapted from the network pictured in van Iersel et al. (2009, Fig. 10), which was constructed from a data set of the yeast *Cryptococcus gattii* (see Hagen et al. 2013, for a following up study). Both networks display the same collection of evolutionary trees (pictured in the Supplementary Material available on Dryad (http://dx.doi.org/10.5061/dryad.f6n8s)), and therefore the same triplets, but they are not equivalent as networks. This is of importance since it implies that even if *all* of the building blocks for some reticulate evolutionary history were to be taken as the input for any given network building method, the method might still output an incorrect history.

To address this problem, it was recently proposed that networks be constructed using a network analog of triplets called *trinets* (Huber and Moulton 2012). Trinets are rooted phylogenetic networks with three leaves (see e.g., Fig. 1 (iii)–(v)); they can be induced on any three leaves of a rooted network by taking the union of all paths from the root to one of the three leaves, and then removing all vertices that lie above the last vertex that is on all such paths, and suppressing parallel edges (see Supplementary Material available on Dryad (http://dx.doi.org/10.5061/dryad.f6n8s) for an illustration). For example, in Figure 1 the trinet pictured in (iii) is induced on the three leaves c,e,f of the network pictured in (i). Note that in this example, even though the two networks in (i) and (ii) both induce the trinet pictured in (v), the trinets (iii) and (iv) that they induce on c,e,f are not equivalent. In particular, it follows that the networks in (i) and (ii) are also not equivalent. Thus, considering trinets could hold some promise for distinguishing between networks, especially since some special types of rooted phylogenetic networks (e.g., level-1, level-2, and tree-child) are in fact encoded by their trinets (see e.g.,Huber and Moulton 2012; van Iersel and Moulton 2014).

Even so, when trying to extend these results on trinet encodings to more general networks we were somewhat surprised to discover that trinets do not necessarily encode networks. Indeed, more generally, in this article we shall show that even if we are given the networks induced on *all* subsets of the leaves of a network except for the leaf-set itself, (which includes all possible trinets), we still do not necessarily have enough information to encode the network. More specifically, for any set X of taxa of size at least three, we shall present an example of two nonequivalent rooted, binary phylogenetic networks with leaf set X which both induce exactly the same network on any subset Y of X with Y≠X (see Theorem 2). As an illustration, we present these two networks in the case that X has four elements in Figure 2. In addition, in the Supplementary Material available on Dryad (http://dx.doi.org/10.5061/dryad.f6n8s), we show that these networks also induce exactly the same set of evolutionary trees. Hence, even knowing all of the induced networks together with all of the induced trees for each of these two networks is still not enough information to distinguish between them.

**Figure 2.**
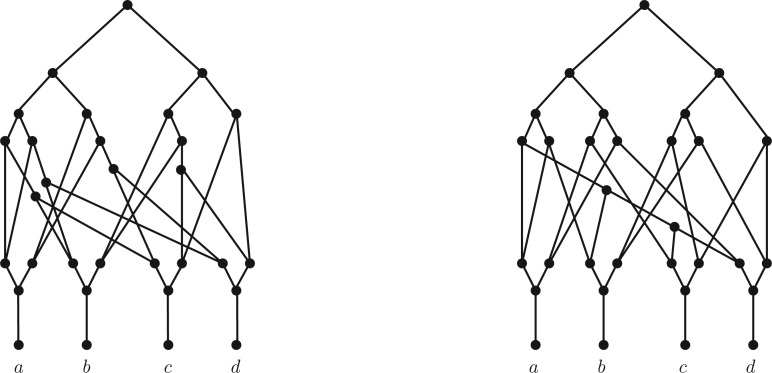
Two distinct rooted phylogenetic networks on the set of taxa {a,b,c,d}. The networks induce exactly the same set of trinets (pictured in the Supplementary Material available on Dryad (http://dx.doi.org/10.5061/dryad.f6n8s)) and also the same set of trees.

Our examples were inspired by some results due to Thatte concerning the reconstructability of the so-called pedigree graphs (Thatte 2008), which are used to represent ancestral relationships between individuals in a population. Thatte was able to show that a pedigree cannot in general be reconstructed from the collection of its proper subpedigrees. Although this result is similar in nature to ours, it is not a simple corollary, as pedigree graphs have quite a different structure to phylogenetic networks (e.g., a pedigree graph can have multiple roots or “founders” and all other vertices have two parents). Moreover, Thatte's concept of a subpedigree is different from our concept of a network induced on a subset of a network's leaves. Intriguingly, both Thatte's and our results are somewhat related to the Kelly–Ulam reconstruction conjecture that states that a graph is uniquely determined by all of its subgraphs. This conjecture is still open, although for directed graphs it is known to be false (see e.g., Stockmeyer 1977). Even so there are again important mathematical distinctions between graphs in general and phylogenetic networks and pedigrees (e.g., graphs are not labeled by a set of taxa and the concept of a subgraph is different from an induced network).

The contents of the rest of the article are as follows. First we present some mathematical preliminaries on phylogenetic networks and also some terminology concerning binary sequences which will be key for constructing our examples. Then, given any leaf set of size at least three we present an example of two distinct *nonbinary*, rooted phylogenetic networks having the same leaf set which both induce exactly the same network on any proper subset of their leaves. These were the first examples that we discovered, and at the time we were uncertain as to whether or not there could be examples of binary networks with this property, as there are various mathematical results in phylogenetics that hold for binary trees/networks but not for nonbinary ones. However, by adapting our nonbinary networks we are also able to construct two binary networks with the same property. Since the proof of this fact follows the same approach to that for the nonbinary case but is considerably more technical, we shall present this in the Appendix. We conclude with a brief discussion of some ramifications and future directions as well as some potential consequences of our results for constructing reticulate evolutionary histories.

## Mathematical Definitions

### Digraphs

The basic graph-theoretical structure that underlies the phylogenetic networks in this article is called a *digraph*. This is a connected, directed graph G consisting of a set of vertices V(G) representing taxa (both hypothetical and sampled) and a set E(G) of directed edges or *arcs* that join pairs of them. We denote an arc starting at vertex u and ending at vertex v by (u,v), and call u a *parent* of v and v a *child* of u. This represents the fact that u is a direct ancestor of v. The *in-* and *outdegree* of a vertex v in G is the number of arcs ending and starting at v, respectively. A vertex of G that has outdegree 0 is called a *leaf* of G (which corresponds to a sampled taxon, either extinct or extant), and the set of all leaves of G is denoted by L(G). Note that vertices with indegree at most one and outdegree at least two represent speciations, whereas those with indegree at least two represent reticulations (e.g., evolutionary events such as hybridization and recombination). If a digraph G has a unique vertex with indegree zero, corresponding to a common ancestor of all of the taxa in question, then that vertex is called the *root* of G, denoted by ρ(G), and we call G a *rooted digraph*. If G is rooted and $G^{\prime }$ is a further rooted digraph then we say that G and $G^{\prime }$ are *isomorphic (as digraphs)* if they are isomorphic in the usual graph-theoretical sense. If, in addition to being isomorphic, every leaf is mapped to itself by the underlying map, then G and $G^{\prime }$ are called *equivalent*.

A digraph with no directed cycles is called a *directed acyclic graph (DAG)*. For a rooted DAG G, a vertex in G that is neither a leaf nor the root is called an *interior vertex* of G. In addition, a vertex u in G is called an *ancestor* of a vertex v in G if u and v are equal (Although this means that in mathematical terms every vertex is considered to be an ancestor of itself, we adopt this mathematical convention as it simplifies the mathematics and is a common assumption in the theory of directed graphs.) or there exists a directed path in G starting at u and ending at v. If u is an ancestor of v but u=v then we say that v is *below* u. Thus, in a DAG a vertex v can never be below itself, which corresponds to the fact that v cannot be a biological descendent of itself. Furthermore, if G has at least three vertices and v is a vertex with outdegree one then we call v *degenerate* if indegree of v is not at least two. Finally, we call G *binary* if the outdegree of ρ(G) is two and the sum of the indegree and outdegree of every interior vertex of G is three.

### Phylogenetic Trees and Networks

Suppose for the remainder of the article that X is some (nonempty) set of taxa. A *(phylogenetic) network* N *(on* X*)* is a rooted DAG without degenerate vertices whose set of leaves is X. Unless the phylogenetic network N in question has precisely two vertices, we always assume that the outdegree of the root of N is at least two. Note that a network N that does not contain vertices with indegree two or more is just an evolutionary or *phylogenetic tree (on* X*)*. As usual, we call a phylogenetic tree in which every leaf is the child of the root a *star tree*.

Now, suppose that Y is a nonempty subset of the set X of species. We now consider the subnet of N induced by restricting our attention to the leaves in Y. The *lowest stable ancestor* lsa(Y) *of* Y *in* N is the vertex w∈V(N)−X that lies on *all* directed paths from the root ρ(N) of N to the elements in Y, so that no vertex of N below w enjoys this property. In case lsa(X)=ρ(N), we call N *recoverable*. The *subnet* $N{|}_{Y}$ of N induced by Y is defined as the phylogenetic network on Y obtained from N as follows: First, delete all vertices of N (and their incident arcs) that are not on a directed path from lsa(Y) to some element in Y. Next, repeatedly *suppress* all resulting degenerate vertices (i.e. replace any such vertex v and the two arcs (u,v) and (v,w) containing it by a single arc (u,w)) and remove all parallel arcs until a phylogenetic network on Y is obtained. This definition for a subnet was introduced by Huber and Moulton (2012), and it aims to capture features that can be recovered from data (e.g., all degenerate vertices are suppressed as it would not be possible to decide how many degenerate vertices to include in a reconstructed network). Note that $N{|}_{X}=N$ if and only if N is recoverable. Also note that every subnet of N induced by restricting N to some nonempty subset of its leaves is necessarily recoverable.

We say that two phylogenetic networks N and $N^{\prime }$ on X are *network-equivalent* if for every nonempty, proper subset Y of X, the phylogenetic networks $N{|}_{Y}$ and $N^{\prime }{|}_{Y}$ are equivalent. Thus, two phylogenetic networks are equivalent if and only if they represent the same evolutionary histories. Note that the following useful observation concerning network-equivalence is an immediate consequence of our definitions.

**Lemma 1.** *Suppose that* $N_{1}$ *and* $N_{2}$ *are two recoverable phylogenetic networks on* X. *If* $N_{1}$ *and* $N_{2}$ *are equivalent, then* $N_{1}$ *and* $N_{2}$ *are network-equivalent*.

### Binary Sequences

All of our networks will be constructed using special types of binary sequences, that is, sequences over the alphabet {0,1}. We use binary sequences since they provide a convenient way to encode the vertices of certain phylogenetic trees that will be relevant to our constructions.

As our examples rely on using some special types of binary sequences we now introduce some general terminology concerning such sequences. Suppose that n is a nonnegative integer. We denote by l(w) the *length* of a binary sequence w. We let ∅ denote the *empty sequence*, that is, the unique sequence with length 0, let $w_{k,n}$ be the binary sequence of length n with 0's in all but the k-th place, 1≤k≤n, and let $0_{n}$, $1_{n}$ be the binary sequences of length n consisting of all 0's and all 1's, respectively. We also let $B_{n}$ denote the set of all binary sequences that have length n. Note that $B_{0}=\{\emptyset \}$.

Now, assume n≥1. For each sequence w in $B_{n}$ and all 1≤i≤n, we denote by ${\left[w\right]}_{i}$ the i-th letter of w starting from the left. We define the *weight* of w as $\sum_{i=1}^{n}{\left[w\right]}_{i}$, that is, the number of 1's contained in w. Moreover, for each sequence $w\in B_{n},$ we define the *support* supp(w) of w to be the subset of {1,2,…,n} consisting of all indices i∈{1,…,n} with ${\left[w\right]}_{i}=1$. Finally, we denote by $B_{n}^{1}$ and $B_{n}^{2}$ the subsets of $B_{n}$ consisting of sequences whose weights are odd and even, respectively. Note that we will assume that $0_{n}$ is the only sequence in $B_{n}$ that is contained in neither $B_{n}^{1}$ nor $B_{n}^{2}$. Thus, $|B_{n}^{1}|=2^{n-1}$ while $|B_{n}^{2}|=2^{n-1}-1$. As an illustration of these definitions, $w_{2,3}=010$, the weight of the sequence 011 is 2 and its support is {2,3}, and $B_{3}^{1}=\{001,010,100,111\}$, $B_{3}^{2}=\{110,101,011\}$.

Now, suppose that w and $w^{\prime }$ are two binary sequences. Then $w^{\prime }$ is called a *prefix of* w if $l(w^{\prime })\leq l(w)$ and ${\left[w^{\prime }\right]}_{i}={\left[w\right]}_{i}$ holds for all $1\leq i\leq l(w^{\prime })$. Note that the empty sequence is a prefix of every binary sequence. Also, if B is a set of binary sequences and w∈B, then we call a sequence $w^{\prime }\in B-\{w\}$ a *precursor of* w *(in* B*)* if $w^{\prime }$ is a prefix of w, that is, a prefix of w is a precursor of w in B if and only if this prefix is contained in B−{w}. And if, in addition, every precursor of w in B other than $w^{\prime }$ is also a precursor of $w^{\prime }$ in B, then we say that $w^{\prime }$ is the *maximal precursor of* w *(in* B*)*. Note that if this exists, then it is unique. Finally, we call w a *common precursor of* B if, for every sequence $w^{\prime }\in B-\{w\}$, w is a precursor of $w^{\prime }$ in B.

## Main Results

### Nonbinary Network Examples

In this section we shall present two nonbinary, phylogenetic networks $N_{1}$ and $N_{2}$ on an arbitrary set X with at least three elements that are not equivalent and prove that they are network-equivalent.

**Figure 3.**
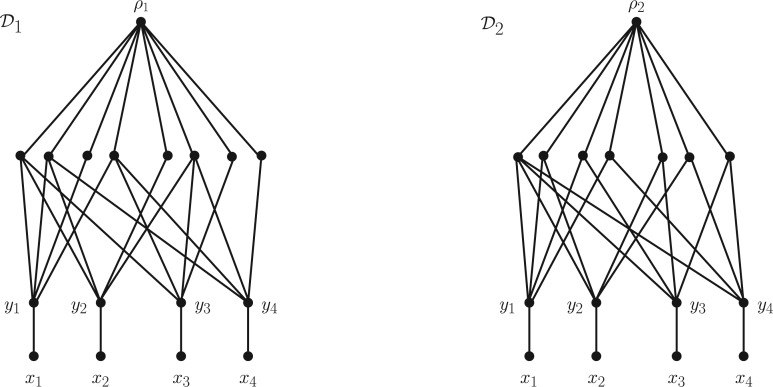
The rooted DAGs $D_{1}$ and $D_{2}$ for the case n=4 and $X=\{x_{1},x_{2},x_{3},x_{4}\}$. The labels of the vertices in $B_{4}^{i}$, i=1,2, directly below the root in both DAGs are omitted; listed from left to right they are 1110,1101,1000,1011,0100,0111,0010,0001 for $D_{1}$, and 1111,1100,1010,1001,0110,0101,0011 for $D_{2}$.

We begin by defining two rooted DAGs $D_{1}$ and $D_{2}$ from which we will obtain $N_{1}$ and $N_{2}$, respectively. Let n≥3, let $X=\{x_{1},\ldots ,x_{n}\}$, and let $Y=\{y_{1},\ldots ,y_{n}\}$ be a set such that X∩Y=∅. For i=1,2, associate to X and Y the rooted DAG $D_{i}$ with vertex set $X\cup Y\cup B_{n}^{i}\cup \{\rho_{i}\}$, and arc set comprising of (i) for all $u\in B_{n}^{i}$ the arcs $\left(\rho_{i},u\right)$, (ii) for all 1≤j≤n the arcs $\left(y_{j},x_{j}\right)$, and (iii) for all 1≤j≤n and $u\in B_{n}^{i}$ the arcs $\left(u,y_{j}\right)$ if and only if ${\left[u\right]}_{j}=1$. Note that X is the set of leaves of $D_{i}$ and $\rho_{i}$ is the root. We illustrate these DAGs for n=4 in Figure 3 and list the binary sequences that label the vertices in $B_{n}^{1}$ and $B_{n}^{2}$ in its caption. To obtain the phylogenetic networks $N_{1}$ and $N_{2}$ we just suppress all degenerate vertices of $D_{1}$ and $D_{2}$, respectively. Note that both $N_{1}$ and $N_{2}$ are recoverable because we have lsa$(X)=\rho_{1}$ in $N_{1}$ and lsa $(X)=\rho_{2}$ in $N_{2}$.

We now prove the first of our main results.

**Theorem 1** *For every* n≥3, *the networks* $N_{1}$ *and* $N_{2}$ *are not equivalent. However*, $N_{1}$ *and* $N_{2}$ *are network-equivalent*.

*Proof*. To see that $N_{1}$ and $N_{2}$ are not equivalent note that $B_{n}^{1}\cap B_{n}^{2}=\emptyset$ and that sequence $1_{n}$ is contained in $B_{n}^{1}\cup B_{n}^{2}$. Consequently, there exists a child of the root of $N_{1}$ or $N_{2}$ (but not both) that has outdegree n. Thus, $N_{1}$ and $N_{2}$ cannot be equivalent.

We next show that $N_{1}$ and $N_{2}$ are network-equivalent. Let k∈{1,…,n} and put $X^{k}=X-\{x_{k}\}$, $Y^{k}=Y-\{y_{k}\}$. Note that $N_{1}{|}_{X^{k}}$ and $N_{2}{|}_{X^{k}}$ are recoverable as they are subnets of $N_{1}$ and $N_{2}$, respectively. In view of Lemma 1, it therefore suffices to show that $N_{1}{|}_{X^{k}}$ and $N_{2}{|}_{X^{k}}$ are equivalent.

To this end, for any k∈{1,…,n}, we associate for i=1,2 a rooted DAG $D_{i}^{k}$ to $D_{i}$ with the leaf $x_{k}$ removed. In particular, we define $D_{i}^{k}$ to be the rooted DAG with leaf set $X^{k}$ obtained from $D_{i}$ by first deleting all arcs from $D_{i}$ that do not lie on a path from the root $\rho_{i}$ of $D_{i}$ to a leaf in $X^{k}$ and then removing all resulting isolated vertices. Note that $X^{k}\cup Y^{k}\cup \{\rho_{i}\}\subseteq V(D_{i}^{k})\subseteq X^{k}\cup Y^{k}\cup \{\rho_{i}\}\cup B_{n}^{1}-\{w_{k,n}\}$.

For brevity, for the rest of this proof, we let $V_{i}^{k}$ and $E_{i}^{k}$ denote the vertex set and edge set of $D_{i}^{k}$, respectively, and we put $w_{k}=w_{k,n}$. We define a map $\chi_{k}$ from $V_{1}^{k}$ to $V_{2}^{k}$ as follows. Let $\phi_{k}=\phi_{k,n}$ denote the map from $B_{n}$ to $B_{n}$ that “flips” precisely the k-th letter of a sequence in $B_{n}$, that is the map given by, for all $w\in B_{n}$, putting ${\left[\phi_{k}(w)\right]}_{j}={\left[w\right]}_{j}$ for j∈{1,…,n}−{k}, and ${\left[\phi_{k}(w)\right]}_{j}=1-{\left[w\right]}_{j}$ for j=k. Note that $w_{k}\in B_{n}^{1}$ and $supp(\phi_{k}(w_{k}))=\emptyset$. Moreover, the map $\phi_{k}$ induces a bijection ${\bar{\phi }}_{k}$ from $B_{n}^{1}-\{w_{k}\}$ to $B_{n}^{2}$. Using this bijection, we now define the map $\chi_{k}$ by putting, for $v\in V_{1}^{k}$,

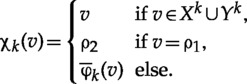

We shall show that this map is a bijection from $V_{1}^{k}$ to $V_{2}^{k}$ that extends to an isomorphism from $D_{1}^{k}$ to $D_{2}^{k}$ which maps every element in $X^{k}$ to itself. This implies that $N_{1}{|}_{X^{k}}$ and $N_{2}{|}_{X^{k}}$ are equivalent.

Clearly, $\chi_{k}$ maps every element in $X^{k}$ to itself. To see that $\chi_{k}$ is a bijection, note that since $w_{k}$ is the only element in $B_{n}^{1}$ that is contained in $V(D_{1})$ but not $V_{1}^{k}$, we have $B_{n}^{1}-\{w_{k}\}\subseteq V_{1}^{k}$. Combined with the fact that $B_{n}^{2}\subseteq V_{2}^{k}$ also holds and that ${\bar{\phi }}_{k}$ is a bijection, it follows that $\chi_{k}$ is a bijection.

To see that $\chi_{k}$ induces an isomorphism between $D_{1}^{k}$ and $D_{2}^{k}$ it suffices to show for all $v,w\in V_{1}^{k}$, that $(v,w)\in E_{1}^{k}$ if and only if $(\chi_{k}(v),\chi_{k}(w))\in E_{2}^{k}$. In view of $\chi_{k}(u)=u$ holding for all $u\in V_{1}^{k}-B_{n}^{1}$ and $\left(\rho_{i},w)\in E(D_{i}\right)$ holding for all $w\in B_{n}^{i}$, it follows that we may restrict our attention to showing that for all j∈{1,…,n}−{k} and all $w\in B_{n}^{1}-\{w_{k}\}$ we have that $(w,y_{j})\in E_{1}^{k}$ if and only if $(\chi_{k}(w),\chi_{k}(y_{j}))\in E_{2}^{k}$. So let j∈{1,…,n}−{k} and $w\in B_{n}^{1}-\{w_{k}\}$. Assume first that $(w,y_{j})\in E_{1}^{k}$. Then ${\left[w\right]}_{j}=1$ and so ${\left[\chi_{k}(w)\right]}_{j}={\left[{\bar{\phi }}_{k}(w)\right]}_{j}=1$ as k=j. Thus, $(\chi_{k}(w),\chi_{k}(y_{j}))=(\chi_{k}(w),y_{j})\in E_{2}^{k}$ as $\chi_{k}(y_{j})=y_{j}$. Conversely, assume that $(\chi_{k}(w),\chi_{k}(y_{j}))\in E_{2}^{k}$. Then since $\chi_{k}(y_{j})=y_{j}$ we have ${\left[{\bar{\phi }}_{k}(w)\right]}_{j}={\left[\chi_{k}(w)\right]}_{j}=1$, and, hence ${\left[w\right]}_{j}=1$ in view of j=k. Thus, $(w,y_{j})\in E_{1}^{k}$, as required.    ▪

### Binary Network Examples

We now extend the definitions of the networks $D_{1}$ and $D_{2}$ defined in the previous section so as to define two binary phylogenetic networks $H_{1}$ and $H_{2}$ that are not equivalent, but which are network-equivalent. We shall just present the definitions of $H_{1}$ and $H_{2}$; the proof of their network-equivalence is quite technical and can be found in the Appendix.

**Figure 4.**
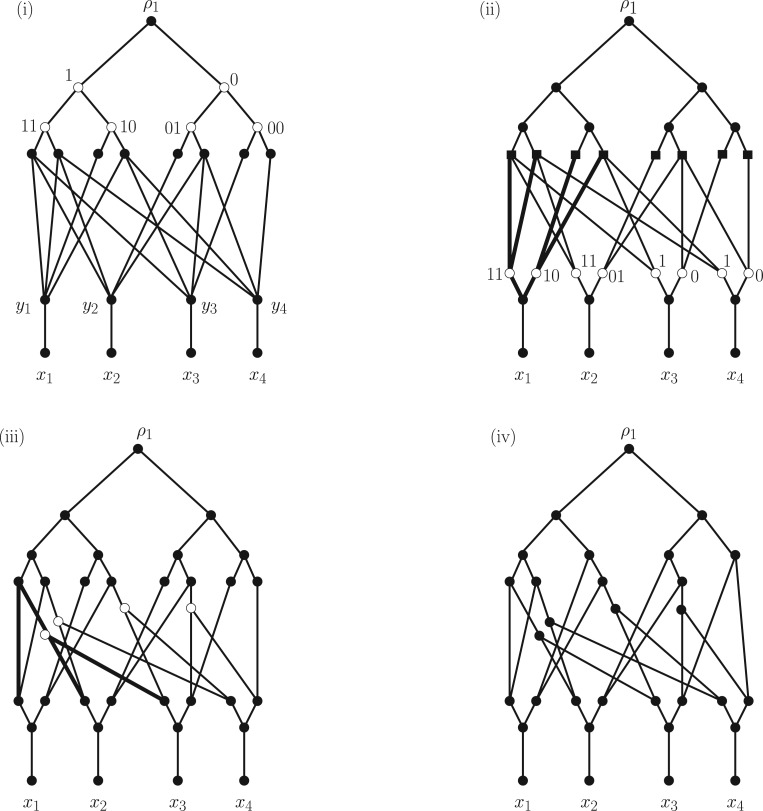
Constructing the network $H_{1}$ from the network $D_{1}$ in the case |X|=4. At each stage, we indicate those vertices that have been inserted by unfilled circles. i) The network $D_{1,1}$ in which the root has been replaced by the tree $P_{4}$. ii) The network $D_{1,2}$ with the vertices in the middle layer indicated by squares. The tree $R_{1}$ is indicated in bold. iii) The network $D_{1,3}$. The tree $C_{supp(w)}$ associated to the binary sequence w=1110 is indicated in bold. iv) The network $H_{1}$ obtained by suppressing all vertices in $D_{1,3}$ having indegree and outdegree equal to one.

Let n≥3 and i∈{1,2}. Starting with the rooted DAGs $D_{i}$ defined in the previous section, we shall define a sequence of three rooted DAGs all having leaf set X, the last one of which will yield $H_{i}$. We illustrate this process in Figure 4, for the rooted DAG $D_{1}$ depicted in Figure 3.

**Figure 5.**
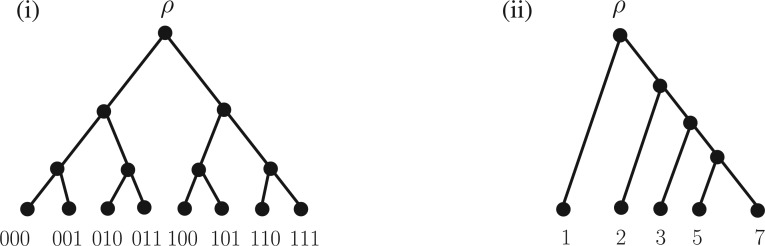
i) The tree $P_{3}$ on $B_{3}$. ii) The rooted caterpillar $C_{\left\{1,2,3,5,7\right\}}$.

*Step 1*: We begin by replacing the star tree containing the root vertex of $D_{i}$ by a tree with leaf set $B_{n}^{i}$ that is a subtree of a certain tree $P_{n}$ which is defined as follows. Let $A_{n}$ be the set of all binary sequences with length at most n. The tree $P_{n}$ is the rooted tree with vertex set $A_{n}$ and arc set consisting of all pairs $(w,w^{\prime })\in A_{n}\times A_{n}$ for which w is the maximal precursor of $w^{\prime }$ in $A_{n}$. Note that the common precursor of $A_{n}$ is clearly the root of $P_{n}$. In addition, since each sequence $w\in A_{n}$ is the maximal precursor of exactly two sequences in $A_{n}$ if w∈Bn, and is not the maximal precursor of any sequence in $A_{n}$ if $w\in B_{n}$, it follows that $P_{n}$ is a binary phylogenetic tree on $B_{n}$. We depict the tree $P_{3}$ in Figure 5(i).

Now, we replace the subgraph of $D_{i}$ with vertex set consisting of the root $\rho_{i}$ of $D_{i}$ and the children of $\rho_{i}$ (i.e. the star tree on $B_{n}^{i}$} with root $\rho_{i}$) by the (necessarily binary) restriction $P_{n}{|}_{B_{n}^{i}}$ of $P_{n}$ to $B_{n}^{i}$. Let $D_{i,1}$ denote the resulting rooted DAG (see e.g., Fig. 4(i)).

*Step 2*: We now replace each of the vertices $y_{j}$, 1≤j≤n, in the “bottom layer” of $D_{i,1}$ by a tree $R_{j}$. This tree is defined by reversing the direction of all arcs in the tree obtained by restricting the tree $P_{n}$ to the set $B_{n,j}^{i}$ of all binary sequences in $B_{n}^{i}$ whose j-th letter is 1. Note that the unique leaf of $R_{j}$ is $y_{j}$ since for all 1≤j≤n the source set of $R_{j}$ is $B_{n,j}^{i}$ and $\overset{n}{\underset{j=1}{\cup }}B_{n,j}^{i}=B_{n}^{i}$ which is the leaf set of $P_{n}{|}_{B_{n}^{i}}$.

Now, note that, for all 1≤j≤n, the indegree of $y_{j}$ in $D_{i}$ is $|B_{n,j}^{i}|=2^{n-2}$ and that therefore the indegree of $y_{j}$ in $D_{i,1}$ is also $2^{n-2}$. We now replace for all 1≤j≤n the subgraph of $D_{i,1}$ induced on the set $\{y_{j}\}\cup B_{n,j}^{i}$ by $R_{j}$. Let $D_{i,2}$ denote the resulting rooted DAG (see e.g., Fig. 4(ii)).

*Step 3*: The final stage of the construction involves replacing each of the vertices in the “middle layer” of $D_{i,2}$ with another phylogenetic tree which is defined as follows.

Let $A=\{a_{1},\ldots ,a_{k}\}$, k≥1, denote a set of positive integers with $a_{1}<\ldots <a_{k}$. If k=1, then we denote by $C_{A}$ the phylogenetic tree whose unique leaf is labeled by the sole element in A. More generally, for k≥2 we denote by $C_{A}$ the (up to equivalence) unique binary phylogenetic tree on A such that, over all non-leaf vertices v of $C_{A}$, the collection of leaves below v is $\cup_{1\leq j<k}\left\{\left\{a_{j},a_{j+1},\ldots ,a_{k}\right\}\right\}$. Note that $C_{A}$ is an example of a rooted caterpillar tree (see e.g., Semple and Steel 2003). In Figure 5(ii) we present the tree $C_{A}$ for A={1,2,3,5,7}.

Now, any nondegenerate vertex w of $D_{i,2}$ is not binary if and only if $w\in B_{n}^{i}$ and |supp(w)|>2. Therefore, we shall consider vertices in $B_{n}^{i}$ whose support has size at least three. We shall replace all such vertices w by a rooted tree that is derived from the tree $C_{supp(w)}$ as follows (essentially, we replace w and its outgoing arcs by a rooted caterpillar whose leaves are the children of w). Put $Y_{w}:=\{y_{t}\in Y:t\in supp(w)\}$. Then, since for all 1≤j<l≤n the trees $R_{j}$ and $R_{l}$ defined in Step 2 do not share an interior vertex in $D_{i,2}$, it follows that for every child $w^{\prime }$ of w there exists a unique vertex $y\in Y_{w}$ below $w^{\prime }$. For all t∈supp(w) let $a_{t}$ denote the child of w in $D_{i,2}$ that lies on the path from w to $y_{t}$ so that, in particular, the set of children of w is $\left\{a_{t}:t\in supp(w)\right\}$. To obtain the final digraph $D_{i,3}$ in our sequence, we replace, for each $w\in B_{n}^{i}$ with |supp(w)|>2, the subgraph of $D_{i,2}$ induced on the set consisting of w and its children by the tree obtained from $C_{supp(w)}$ by replacing each of its leaves t∈supp(w) by the corresponding child $a_{t}$ of w and replacing its root by w (see e.g., Fig. 4(iii)).

The phylogenetic network $H_{i}$ is now defined to be the rooted DAG obtained from $D_{i,3}$ by suppressing all degenerate vertices (see e.g., Fig. 4(iv)). Note that, by construction, the leaf set of $H_{i}$ is X and $H_{i}$ is binary. Also note that $H_{i}$ is recoverable.

The proof of our second main result is quite technical and is given in the Appendix.

**Theorem2** *For every* n≥3, *the binary phylogenetic networks* $H_{1}$ *and* $H_{2}$ *are not equivalent. However*, $H_{1}$ *and* $H_{2}$ *are network-equivalent*.

## Discussion

Our examples illustrate a problem with generalizing evolutionary models from rooted trees to rooted networks. We show that there are pairs of phylogenetic networks on an arbitrary set of taxa that are not equivalent, and yet display the same set of evolutionary trees (see Supplementary Material available on Dryad (http://dx.doi.org/10.5061/dryad.f6n8s) for additional details), as well as the same set of induced subnetworks. Although these examples are artificial in their construction, they still point to the possibility that this phenomenon could arise in nature, especially since phylogenetic networks can be extremely complex (see e.g., Kunin et al. 2005; Dagan et al. 2008).

The problem that we have presented has some potential ramifications for the development and use of new methods for constructing networks that explicitly represent evolution. First, as mentioned in the introduction, it implies that in practice we will have to be careful to ensure that the output from any network construction method is uniquely determined by its input. This in itself is not necessarily a great problem since even when we construct phylogenetic trees there can be multiple solutions (e.g., there can be several most parsimonious trees). Second, given that we know that there are cases where a network cannot be uniquely recovered from all of its induced subnetworks, it becomes important to characterize under which conditions these cases will be manifested, and we should try to understand how often biological data will actually meet these conditions. Finally, in the context of extending consensus and supertree methods to include phylogenetic networks, our result shows that, unlike trees, it will not be possible to develop supernetwork methods in general that are consistent, that is methods that are guaranteed to output a given network from all of its induced subnetworks. However, again it will be interesting to better understand how important this will actually be in practice.

Even though we have found that networks are not necessarily encoded by their induced subnets in general, some classes of networks are. For example, level-2 networks and thus also phylogenetic trees and level-1 networks are encoded by their trinets (note that the level of a binary phylogenetic network is the maximum number of indegree-2 vertices taken over all biconnected components of the network) (van Iersel and Moulton 2014). Hence, it might be of interest to determine which types of networks are encoded by their induced subnets and also to possibly concentrate on developing methods to construct these special types of networks. Note that various methods have already been designed to construct special types of networks (see e.g., Willson 2012), but this obviously requires some care to ensure that the properties of the networks under consideration are realistic enough to represent real data. Note also that the level of the networks in our examples is exponential in |X| (it is $(2^{n-2}-1)n$ with n=|X|). Hence, it could be of interest to decide whether networks with reasonably low level relative to the size of their leaf set (e.g., linear level as function of |X|) are encoded by their subnets.

Even if we are not necessarily able to encode a network by its subnets, it could still be of interest to investigate whether at least some parameters (e.g., the number of reticulation vertices) can be determined or at least approximated by the knowledge of their induced subnets (or even trees). In addition, it could be useful to decide whether or not networks might be encoded if more information is available (e.g., if we are given branch lengths/dates for vertices or some model of evolution). Note that Thatte and Steel investigated reconstructability of pedigrees assuming a certain probabilistic model and were able to prove some encoding results for pedigrees in general (see e.g., Thatte and Steel 2008; Thatte 2013), so analogous results might also hold for phylogenetic networks.

There are some related mathematical problems that are also worth mentioning. It has been shown that a graph drawn uniformly at random is encoded by its subgraphs with probability 1, as the size of the vertex set goes to infinity (see e.g., Bollobás 1990). It would be interesting to work out the probability that a randomly selected phylogenetic network is encoded by its induced subnets. This might also provide some clues about whether or not networks arising in practice could be expected to be encoded by induced subnets or not. In particular, the aforementioned probabilistic result suggests that maybe networks on large sets of taxa that are not encoded by their induced subnetworks might be quite rare in practice. In addition, an interesting algorithmic question is the following: if we are given a phylogenetic network, can we decide efficiently if it is uniquely encoded by its induced subnets? And, if we are given a set of networks, can we efficiently decide if they are induced subnets of some network?

In conclusion, even if there may be more than one network that can induce the same set of trees and/or subnetworks, it is still useful to find ways to construct these networks so that alternative evolutionary scenarios can be explored. This has already proven a useful strategy in phylogenetics (for example, understanding the number of reconciliations of a gene tree with a species tree (Bansal et al. 2013)). In regards to this, it would be interesting to develop ways to determine how many networks can potentially display the same set of subnetworks. More generally, a better understanding of the structure of networks in terms of substructures could also give us a better understanding of the performance of current methods for network construction, and will hopefully also eventually help us to design new methods for confidently recovering reticulate evolutionary histories.

## Appendix

In this appendix we prove Theorem 2. Assume that n is a positive integer and that k∈{1,…,n}. We will use the following lemmas concerning the trees $P_{n}$ and $C_{A}$ used in the construction of $H_{1}$ and $H_{2}$. For brevity, for any sequence $w\in B_{n}$ with nonempty support (with the natural order), we put $C_{w}=C_{supp(w)}$. Also and as in the proof of Theorem 1, we let $\phi_{k}:B_{n}\to B_{n}$ be the map that “flips” precisely the k-th letter of a sequence in $B_{n}$.

**Lemma 2.** *For each sequence* $w\in B_{n}-\{0_{n},w_{k,n}\}$, *the trees* $C_{w}{|}_{supp(w)-\{k\}}$ *and* $C_{\phi_{k}(w)}{|}_{supp(\phi_{k}(w))-\{k\}}$ *are isomorphic*.

*Proof*. Put $w_{k}=w_{k,n}$. Let $w\in B_{n}-\{0_{n},w_{k}\}$. Then neither supp(w)=∅ nor supp(w)={k} holds. This implies that the trees $C_{w}$ and $C_{\phi_{k}(w)}$ are both well-defined. Let $w^{\prime }$ denote the unique sequence in $B_{n}$ whose support is supp(w)−{k}, which is clearly not the empty set. Then the tree $C_{w^{\prime }}$ is also well-defined and, as is straightforward to see, it is isomorphic to $C_{w}{|}_{supp(w^{\prime })}$. In view of ∅=supp(w′)⊆supp(ϕk(w)), we also have that $C_{w^{\prime }}$ is isomorphic to $C_{\phi_{k}(w)}{|}_{supp(w^{\prime })}$. Since $supp(w)-\{k\}=supp(w^{\prime })=supp(\phi_{k}(w))-\{k\}$, it follows that $C_{w}{|}_{supp(w)-\{k\}}$ must be isomorphic to $C_{\phi_{k}(w)}{|}_{supp(\phi_{k}(w))-\{k\}}$.    ▪

We now establish a similar result for the tree $P_{n}$. For $A_{n}$ as defined in Step 1 of the construction, a set $A\subseteq A_{n}$ of binary sequences is called *complete* if it contains a (necessarily unique) common precursor. Given such a set, generalizing the definition of $P_{n}$, we let P[A] denote the directed tree with vertex set A and arc set the set of all arcs $(w,w^{\prime })\in A\times A$ for which w is the maximal precursor of $w^{\prime }$ in A. Note that $P_{n}=P[A_{n}]$.

**Lemma 3.**

*(i) The trees* $P_{n}{|}_{B_{n}^{1}-\{w_{n,k}\}}$ *and* $P_{n}{|}_{B_{n}^{2}}$ *are isomorphic*.
*(ii) For all* j∈{1,…,n}−{k}, *the trees* $P_{n}{|}_{B_{n,j}^{1}}$ *and* $P_{n}{|}_{B_{n,j}^{2}}$ *are isomorphic*.

*Proof*. Let $A^{*}$ be the set of all binary sequences of finite length. For all l≥1, define the map $\psi_{l}:A^{*}\to A^{*}$ by

$$\psi_{l}:A^{*}\to A^{*}:w\mapsto \{\begin{array}{cc} \phi_{l,n}(w) & \text{if}\;w\in B_{n}\;\text{for}\;\text{some}\;l\leq n,\\ w & \text{else.} \end{array}$$

Now, note that if a subset $A\subseteq A_{n}$ is complete, then the set $\psi_{l}(A)$ is complete since, for any two distinct sequences $w,w^{\prime }\in A$, we have that w is a precursor of $w^{\prime }$ in A if and only if $\psi_{l}(w)$ is a precursor of $\psi_{l}(w^{\prime })$ in $\psi_{l}(A)$. Moreover, for l=k the map $\psi_{k}$ induces an isomorphism between the trees P[A] and $P[\psi_{k}(A)]$. In particular, $\psi_{k}$ induces, for every subset $B\subseteq B_{n}$, an isomorphism between $P_{n}{|}_{B}$ and $P_{n}{|}_{\psi_{k}(B)}$.

Both statements in the lemma now follow in view of the fact that $\psi_{l}(B_{n}^{1}-\{w_{k,n}\})=\phi_{l}(B_{n}^{1}-\{w_{k,n}\})=B_{n}^{2}$ holds for all 1≤l≤n and $\psi_{l}(B_{n,j}^{1})=B_{n,j}^{2}$ holds for all 1≤j,l≤n with j=l.

We now prove Theorem 2. We first show that $H_{1}$ and $H_{2}$ are not equivalent. Indeed, let i∈{1,2}. If v is a vertex in $D_{i}$ then the set of leaves below v equals X if and only if v is the root of $D_{i}$ or $v=1_{n}$. Since if m is even $1_{m}\in B_{m}^{2}$ and if m is odd $1_{m}\in B_{m}^{1}$, the number of vertices v in $D_{i}$ for which the set of leaves below v equals X is different in $D_{1}$ and $D_{2}$. Thus, $H_{1}$ and $H_{2}$ cannot be equivalent.

We now prove that $H_{1}$ and $H_{2}$ are network-equivalent. Let $X^{k}=X-\{x_{k}\}$ and $Y^{k}=Y-\{y_{k}\}$. Note that the subnets $H_{1}^{k}=H_{1}{|}_{X^{k}}$ and $H_{2}^{k}=H_{2}{|}_{X^{k}}$ are recoverable as they are subnets of $H_{1}$ and $H_{2}$, respectively. Hence, in view of Lemma 1, it suffices to show that $H_{1}^{k}$ and $H_{2}^{k}$ are equivalent.

Now, put $D_{i,0}=D_{i}$. For 0≤j≤3, let $D_{i,j}^{k}=(V_{i,j}^{k},E_{i,j}^{k})$ denote the rooted DAG with leaf set $X^{k}$ obtained from $D_{i,j}$ by first deleting all arcs from $D_{i,j}$ that do not lie on a path from the root of $D_{i,j}$ to a leaf in $X^{k}$ and then removing the resulting isolated vertices. Note that $D_{i,0}^{k}=D_{i}^{k}$ and that $H_{i}^{k}$ is the phylogenetic network on $X^{k}$ obtained from $D_{i}^{k}$ by suppressing all degenerate vertices.

By Lemma 3(i), there exists a bijection $\chi_{1}^{k}:V_{1,1}^{k}\to V_{2,1}^{k}$ that extends to an isomorphism from $D_{1,1}^{k}$ to $D_{2,1}^{k}$ such that, for all $v\in V_{1,1}^{k}$, we have $\chi_{1}^{k}(v)=\psi_{k}(v)$ if $v\in V_{1,1}^{k}-V_{1,0}^{k}$ and $\chi_{1}^{k}(v)=\chi_{k}(v)$ else where $\chi_{k}$ is the bijection from $V_{1}^{k}$ to $V_{2}^{k}$ given in the proof of Theorem 1. Using Lemma 3(ii), there also exists a bijection $\chi_{2}^{k}:V_{1,2}^{k}\to V_{2,2}^{k}$ that extends to an isomorphism from $D_{1,2}^{k}$ to $D_{2,2}^{k}$ for which $\chi_{2}^{k}(v)=\chi_{1}^{k}(v)$ holds for all $v\in V_{1,1}^{k}$. Moreover, by Lemma 2, there also exists a bijection $\chi_{3}^{k}:V_{1,3}^{k}\to V_{2,3}^{k}$ that extends to an isomorphism from $D_{1,3}^{k}$ to $D_{2,3}^{k}$ for which $\chi_{3}^{k}(v)=\chi_{2}^{k}(v)$ holds for all $v\in V_{1,2}^{k}$. Consequently, $H_{1}^{k}$ and $H_{2}^{k}$ must be equivalent, as required.

## Supplementary material

Data available from the Dryad Digital Repository: http://dx.doi.org/10.5061/dryad.f6n8s.
